# Innovations in teaching during the COVID-19 pandemic: comparisons of the impacts of different teaching approaches in psychiatric nursing on undergraduate nursing students

**DOI:** 10.1186/s12909-023-04819-8

**Published:** 2023-11-03

**Authors:** Yawei Shan, Xuemei Zhou, Wenwen Qi, Xiao Liu, Chuxian Huang

**Affiliations:** 1 School of Nursing, Shanghai University of Traditional Chinese Medicine, No. 1200, Cailun Road, Shanghai, 201203 China; 2 Department of Nursing, Shanghai Hongkou Mental Health Centre, Shanghai, China; 3 Department of Nursing, Shanghai Mental Health Centre, Shanghai, China

**Keywords:** Academic performance, Education, Psychiatric nursing, Teaching approach, Workload

## Abstract

**Background:**

Psychiatric nursing education was significantly impacted during the COVID-19 pandemic, and innovative teaching can be challenging. This study aims to compare the effectiveness of four approaches to psychiatric nursing education in the context of the pandemic.

**Methods:**

A quasi-experimental design was adopted. Students were subjected to different teaching designs: face-to-face teaching (Class A in 2021), blended teaching with flipped classroom using roleplay (Class B in 2021), live broadcast teaching (Class A in 2022), and online blended teaching with flipped classroom using case studies (Class B in 2022). Multivariable logistic regression was used to analyse the outcomes in terms of academic performance and course workload.

**Results:**

The number of valid data points was 270. The results indicated that compared with Class A in 2021, the two classes in 2022 achieved significantly higher academic performance scores, and Class B in 2021 exhibited a significantly lower workload. Compared with Class A in 2022, Class B in 2021 exhibited a significantly lower workload. Compared with Class B in 2022, Class B in 2021 exhibited a significantly lower workload and achieved lower academic performance scores.

**Conclusion:**

This study demonstrated that innovative teaching designs for psychiatric nursing offer advantages with regard to either facilitating academic performance or optimizing learners’ task loads. Furthermore, blended learning is a promising teaching approach in the context of the COVID-19 pandemic. Future teaching initiatives could adopt student-centred constructive learning designs and ensure feasible teaching.

**Supplementary Information:**

The online version contains supplementary material available at 10.1186/s12909-023-04819-8.

## Background

In recent years, mental disorders have become a major cause of the global burden pertaining to disease and disability [1]; this increased prevalence has led to a significant increase in the demand for mental health care, thus highlighting the need for nurses to provide quality care to those patients. Therefore, psychiatric nursing education is fundamental for nursing professionals. However, nurses’ undergraduate education generally does not prepare them effectively to provide mental health care because psychiatric nursing has traditionally been perceived as less important and has been viewed as one of the most complex and difficult courses in nursing education [2]. In addition, negative social attitudes towards individuals with mental health disorders or even towards professionals in psychiatric practice constitute one of the main obstacles to learning in these courses [3]. Studies have consistently supported the use of various teaching approaches, such as practical teaching in naturalistic clinical settings or patient simulation, that can enable nursing students to learn and apply knowledge and skills [4]; however, it is difficult to balance teaching requirements with clinical restrictions and the necessary resources to establish a high-fidelity simulation context in which students can practise skills pertaining to at-risk patients in a safe and low-stakes environment [5]. Therefore, psychiatric nursing education has traditionally been conducted in classroom settings with the goals of improving nursing students’ theoretical knowledge, while skills and competency was enhanced during clinical placement [4].

However, the unprecedented global crisis caused by the COVID-19 pandemic impacted nursing education deeply because isolation was considered to be the most common and effective way of preventing infection; thus, traditional teaching activities were hindered due to the higher risk of infection that this situation entailed [6]. Consequently, nursing education is undergoing continuing modification using different approaches in response to changes associated with the pandemic situation and local policies. Due to the rapid development of digital technology and the internet, online education was widely promoted and expanded in higher education during the COVID-19 pandemic, which led to the subversion of conventional teaching methods [7]. Consequently, changes and innovations such as online learning, blended teaching, the flipped classroom, and simulated clinical scenarios continue to challenge nursing education at every level [8]. Explorations of innovative education should consider the factors that might influence different aspects of the acceptability and feasibility of education, such as the prioritization of teaching problems, benefits and risks, the reliability of different approaches, students’ values and preferences, and educational resources [7].

Due to the sudden outbreak and ongoing widespread and intermittent fluctuations of the COVID-19 pandemic, nursing education has become somewhat chaotic due to the suspension of clinical practice and restrictions concerning on-campus teaching. This situation has led researchers to explore viable teaching alternatives to reduce such negative influences, as evidence of effective teaching approaches in this context remains scarce; however, there is no doubt that psychiatric nursing education was significantly impacted by the pandemic, with the most striking such impact being the absence of face-to-face interaction and clinical placement [9]. During this period, several teaching approaches other than traditional classroom teaching emerged, including live broadcast teaching [10], online platform self-directed learning [7] and blended learning with flipped classroom in various modes [5]. However, the reasons for selecting different teaching approaches are diverse, and evidence in this area remains lacking; generally, these reasons are related to the pandemic situation, teaching resources, and teachers’ and students’ preferences, which might influence the quality of teaching.

Live broadcast teaching involves the live digital transformation of courses to facilitate distance learning [10]; this approach was the first to be adopted following the outbreak of the pandemic to substitute for face-to-face lectures when preparations for online teaching platforms and relevant teaching resources were insufficient. Although multimedia teaching based on massive open online courses (MOOCs) was made available on some websites, this approach may not be in line with certain teaching objectives. In addition, MOOCs cannot facilitate real-time dynamic scene display. Due to the development of customized online platforms and multimedia resources, small private online courses (SPOCs) emerged. Moreover, as a substitute for practice teaching, live broadcast demonstrations and seminars were designed, which emphasized students’ acquisition of clinical skills, such as their understanding and development of the skills and techniques needed to support the therapeutic relationship. As measures were implemented to mitigate the effects of the pandemic, teaching design was optimized in the form of blended learning, which is an approach in which online learning is used as a substitute for a portion of face-to-face teaching, and the flipped classroom approach was implemented using approaches such as problem-based case studies or simulation training [11].

However, comparisons of the teaching approaches used for psychiatric nursing are limited, and no consensus regarding a particular approach to teaching has been reached [12, 13]. In other specialties, online teaching (without further classification) has been compared with face-to-face teaching in a number of studies [7]. Generally, basic teaching settings have been divided into synchronous, asynchronous and blended settings. Synchronous teaching (face-to-face, live broadcast) refers to an instructional method that involves real-time interactions between the student and the teacher, while asynchronous teaching (such as video teaching through MOOC or SPOCs) is not constrained by the limitations of time or place [14]. Chen showed that asynchronous teaching (online teaching through video recordings) exhibited no significant differences from face-to-face teaching in terms of academic performance [6], while Kositanurit indicated that asynchronous online education methods can be less effective in medical education [15]. Blended learning with a flipped classroom represents a potential way of enhancing teaching effects through a mixed model featuring both synchronous and asynchronous teaching [11]. As studies on different teaching methods began to emerge during the COVID-19 pandemic and approaches were usually selected in a facilitative way [12], it is worthwhile to explore the effectiveness of different teaching methods, especially with regard to typical synchronous and blended forms of teaching.

Regarding the indicators of the impacts of different teaching approaches in psychiatric nursing, academic performance is the key indicator in the field of education [16], while students’ perceptions of the learning experience constitute an important component of evaluations of the implementation of medical curricula [7], and studies have suggested that higher education institutions should take into account students’ perceived workload with the goal of avoiding the burnout caused by academic pressure and energy consumption when the teaching approaches currently in use become increasingly complex [17]. Therefore, this study aims to report a curriculum design change in terms of the approaches used to deliver psychiatric nursing during the different stages of the COVID-19 pandemic and to verify the following hypotheses: (a) compared to face-to-face teaching, innovative teaching designs of psychiatric nursing offer advantages with regard to either facilitating academic performance or decreasing learners’ task load; (b) teaching effects differ between online synchronous teaching (live broadcast teaching) and blended teaching (blended teaching with flipped classroom, online blended teaching with flipped classroom); and (c) different blended teaching designs have different effects in the context of psychiatric nursing courses.

## Methods

### Design

This study adopted a quasi-experimental design that compared the academic performance and learning task loads of four groups that were subjected to different teaching approaches in 2021 and 2022.

### Participants and sample

A convenience sample of third-year undergraduate nursing students in a baccalaureate degree programme at a Chinese university was recruited. The inclusion criteria were (1) full-time nursing students enrolled in a psychiatric nursing course; (2) having obtained credits for core nursing courses, including health assessment, fundamental nursing and medical nursing; (3) having a sufficiently stable mental state to participate in course learning; and (4) being willing to attend the course tests and report their learning task load. The exclusion criteria were dropouts or individuals undergoing an academic suspension. According to the course selection by students, students were divided into two classes in the spring semester of each year. To confirm the influence of the four teaching approaches, the parameters were set to effect size f (V) = 0.15, α = 0.05, and β = 0.20 using G*Power software [18]. According to the results of the statistical power analysis (linear multiple regression), a minimum sample size of 33 participants in each group was needed.

### Setting and course outline

The selected university has the capacity to admit approximately 130 students each academic year. The psychiatric nursing course is a core competency course as well as a major subject included in China’s national nursing examination. The course focuses on the main mental disorders and disease management by nursing professionals and is delivered over a period of 4 weeks, including 25 credit hours of theoretical teaching and 3 credit hours of practical teaching. The learning objectives were designed in accordance with Bloom’s taxonomy of educational objectives [19].

Due to the COVID-19 pandemic, clinical placements and practical teaching in the psychiatric nursing course were suspended, and shortly after the first class was taught using a face-to-face approach in 2021, a blended teaching approach that featured flipped classroom using roleplay was adopted for the second class in 2021 to reduce exposure to and infection from COVID-19. In 2022, distance teaching was adopted because of the large-scale isolation, and due to students’ requirements, live broadcast teaching was utilized in the first class held during this academic year. For the later class, an online blended teaching approach featuring flipped classroom using case studies was used. To ensure the quality of teaching design and implementation, evidence-based and collaborative lesson planning was performed. Moreover, all iterations of the course included equivalent teaching content, instruction time, duration of required study, compulsory assignments and evaluation items and methods. A SPOC was developed, which featured learning instructions, video-recorded lectures, multimedia information, self-directed quizzes, options to ask/answer questions and online discussions on the digital teaching platform; these materials were available to all students.

### Teaching approaches

#### Face-to-face teaching

The face-to-face teaching approach included 25 credit hours of psychiatric nursing theory; course content was delivered in the form of presentations by instructors. The content of the traditional method is shown in Table 1. The students participated in a three-hour online psychiatric nursing practice session delivered by a nursing practitioner in a mental health centre; the content included an introduction to the environment of a mental health centre as well as common psychiatric crises and ways of managing them. Tencent Meeting software was employed to provide a live broadcast of the online practice.

**Table 1:** Description of the four teaching approaches

Type	Face-to-face teaching	Blended teaching with flipped classroom using roleplay	Live broadcast teaching	Online blended teaching with flipped classroom using case studies
Class	Class A in 2021	Class B in 2021	Class A in 2022	Class B in 2022
Instructor’s role	Presentation and video	Presentation and video; online self-directed learning; flipped classroom (roleplay, reflection, problem-based case study)	Presentation and video via Tencent Meeting	Presentation and video through Tencent Meeting; Online self-directed learning; flipped classroom (problem-based case study)
Digital platform	Online course and exercise	Online course and exercise	Online course and exercise	Online course and exercise
Teaching materials	Course learning instruction	(1) Course learning instruction; (2) Roleplay and reflection instruction; (3) Problem-based learning instruction; (4) Case descriptions and questions	(1) Course learning instruction; (2) Live broadcast app	(1) Course learning instruction; (2) Problem-based learning instruction; (3) Case descriptions and questions
Lessons and teaching strategy	(1) Face-to-face lectures for all sections; (2) Practice: online psychiatric nursing practice	(1) Face-to-face lectures in psychiatric symptomatology, treatment and nursing skills in psychiatric care sections; (2) Self-directed learning on digital platforms in other sections; (3) Flipped classroom (roleplay, reflection and problem-based case study); 5. Practice: online psychiatric nursing practice	(1) Live broadcast lectures for all sections; (2) Practice: online psychiatric nursing practice	(1) Live broadcast lectures in psychiatric symptomatology, treatment and nursing skills in psychiatric care sections; (2) Self-directed learning on digital platforms in other sections; (3) Flipped classroom (problem-based case study); 5. Practice: online psychiatric nursing practice
Assessment	(1) Summative performance with an examination; (2) Medical narrative reflective writing; (3) Learning task load	(1) Summative performance with an examination; (2) Medical narrative reflective writing; (3) Learning task load	(1) Summative performance with an examination; (2) Medical narrative reflective writing; (3) Learning task load	(1) Summative performance with an examination; (2) Medical narrative reflective writing; (3) Learning task load

#### Blended teaching with flipped classroom using roleplay

In line with the integrative review conducted by Rønning and Bjørkly [20], the course combined theoretical lecture-based teaching with self-learning and flipped classroom learning; the course emphasized communication skills and the acquisition of mental health nursing competencies (Table 1). The self-directed learning and flipped classroom learning involved a three-step structured process: preclass self-directed learning and roleplay preparation, in-class readiness tests, roleplay and reflection, and a team-based case study. During the roleplaying exercise, students were divided into groups of approximately five individuals, and the first vignettes pertaining to an anonymized real-world case series in mental health care were described by the teacher before the students engaged in flipped classroom learning. Students could select one case with which they were familiar or in which they were interested to prepare for the roleplay exercise before class. During the flipped classroom teaching, students practised psychotherapeutic communication approaches by engaging in clinical roleplay and reflection under the supervision of by teachers. Students were required to engage in a roleplay exercise featuring patients, therapists, nurses and other members of the patients’ social network. After the roleplaying and reflection, a team-based case study associated with four vignettes was conducted. A total of six cases on the mental health disorders of schizophrenia, depression, bipolar disorder, eating disorder, autism spectrum disorders and attention deficit and hyperactivity disorder were developed.

#### Live broadcast teaching

Similar to the face-to-face teaching approaches described above, the webinar live broadcast theoretical teaching and the online psychiatric nursing practice were conducted using the Tencent Meeting app.

#### Online blended teaching with flipped classroom using case studies

The blended teaching approach with a flipped classroom using roleplay exercises described above was replaced by problem-based learning seminars that were broadcast live using Tencent Meeting. In line with the problem-based learning approach, the seminars were organized by a tutor and focused on problem-solving based on patient scenarios that were the same as the cases used in the clinical roleplay exercises. The learning materials included discussion instructions, case descriptions and relevant questions, which were discussed by students. Students were divided into groups of approximately ten individuals using the broadcast live app function.

### Outcome measures and data collection

The researchers obtained information regarding participants’ credits in core nursing courses through the university’s educational administration system to ensure that participants exhibited the same initial level of achievement before taking the psychiatric nursing course. Sociodemographic characteristics were collected before the course using the online questionnaire platform *Wenjuanxing* (www.wjx.cn). Participants’ of academic performance and perceived workload were assessed at the end of the course as part of the routine assessments of teaching quality. Data regarding participants’ academic performance were collected through course examinations, and their perceived workload was evaluated through *Wenjuanxing*; students were allowed to upload only fully completed questionnaires. Students’ participation in the questionnaire survey was completely voluntary. Data collection was discontinued when no data were uploaded for a period of 7 days.

#### Sociodemographic characteristics

This self-designed questionnaire included items regarding students’ age, gender, birthplace, ethnicity, experience interacting with persons with mental disorders, and history of mental health problems.

#### Grade point average

Participants’ grade point averages (GPAs) before taking the psychiatric nursing course were collected based on the GPA scale constructed by the university. GPA was measured on a 4-point scale, where a score of 4 points represents grades ranging from 95 ~ 100, a score of 3.6 points indicates grades ranging from 90 ~ 94, a score of 3.3 points refers to grades ranging from 85 ~ 89, a score of 3 points represents grades ranging from 80 ~ 84, a score of 2.5 points indicates grades ranging from 75 ~ 79, a score of 2 points refers to grades ranging from 70 ~ 74, a score of 1.5 points represents grades ranging from 65 ~ 69, a score of 1 point indicates grades ranging from ranged 60 ~ 64, and a score of 0 points refers to grades lower than 60. The average score for all courses determined the student’s GPA.

#### Academic performance

Academic performance was assessed through a course exam on theoretical knowledge that was administered one week after the end of the course. The exam contained 30 single-choice questions and 5 glossary explanations that were drawn from the test question bank, which was developed in accordance with the course syllabi and learning objectives. The exam questions were in line with the standards of China’s national nursing examination, and the difficulty levels were determined based on a consensus within the teaching team and passed verifications from the School of Nursing and Teaching Affairs Department of the university. The same number of questions was drawn from each area of teaching content, and the level of difficulty was balanced across the four different classes. The total possible score on the examination was 40.

#### Perceived workload

The perceived workload of courses was measured using the Chinese version of the National Aeronautics and Space Administration Task Load Index (NASA-TLX) [21], which comprises six subscales (or items) pertaining to different aspects of workload (mental demands, physical demands, temporal demands, performance, effort, and frustration). The Chinese version of this index was translated by Liang et al. and included subscales ranging from 0 to 100. The student indicates the numerical score for each of six visual analogue subscales that best matches their experience during the course. The Cronbach’s ɑ coefficient of the Chinese version of the scale was 0.707 [22]. The total mean score of the six subscales reflects the level of perceived workload. The Cronbach’s ɑ coefficient of this scale in this study was 0.864.

### Ethical considerations

This study was strictly guided by the principles of the 2000 Declaration of Helsinki in terms of ethical standards, and the protocol was approved by the Committee on the Ethics of Medical Research of Shanghai Hongkou Mental Health Centre (No. 2021-B06). All study participants provided informed written consent prior to enrolment in the study. The students exposed their private information by participating in the survey; thus, completion of the survey was anonymous, and the confidentiality of the information was ensured. As clinical placement and practical teaching in the psychiatric nursing course were suspended during the COVID-19 pandemic due to measures aimed at combating the epidemic, students underwent one-month clinical practice in the mental health centre during their final year before graduation to enhance their skills in psychiatric nursing.

### Validity and reliability/rigour

This research strongly emphasized quality control in terms of teaching design, implementation, evaluation, learning support and feedback. The course on psychiatric nursing was designed under the guidance of the National Standards for Teaching Quality on Nursing and was implemented by five senior lecturers who either had a Ph.D. or Master’s degree or were advanced practice nurses working in the field of mental health care. The face-to-face teaching pedagogy used for the course had been implemented by the teaching team for five years, while the innovative teaching designs were reviewed, approved and guided by experts on medical education in the university. To ensure the quality of the implementation, the teaching content in the four teaching programmes was delivered by the same teachers on the course team either through face-to-face teaching, video recording or live broadcasting. Furthermore, teaching supervisors provided peer listening and guidance, and the effect of the first round of online teaching based on the newly developed SPOC was tested and published in a peer-reviewed journal [23]. Regarding the quality of the course evaluation, the exam questions were drawn from a test question bank that met the requirements stipulated in the course syllabi, and the difficulty of questions was balanced across different exams. A measurement tool for perceived workload with good validity and reliability was used, and the psychometric properties of this tool are described above.

### Statistical analysis

Statistical analysis was conducted using SPSS 21.0 software (IBM Corp), and *p* < 0.05 (two-tailed) was considered to be significant. Descriptive statistics, including the number (n), percentage (%), mean (M) and standard deviation (SD), were used to analyse participants’ sociodemographic characteristics, academic performance and perceived workload. Normality and equal variance for all the variables were checked by performing the Shapiro–Wilk and Bartlett tests. Multivariable logistic regression was used to control for the confounding impacts of sociodemographic characteristics, and multiple linear regression was used to analyse the influence of the four teaching approaches on students’ academic performance and perceived workload. Variables that could impact teaching outcomes were included in the multivariable models.

## Results

### Participant characteristics and descriptive statistics of the variables

**Fig. 1:**
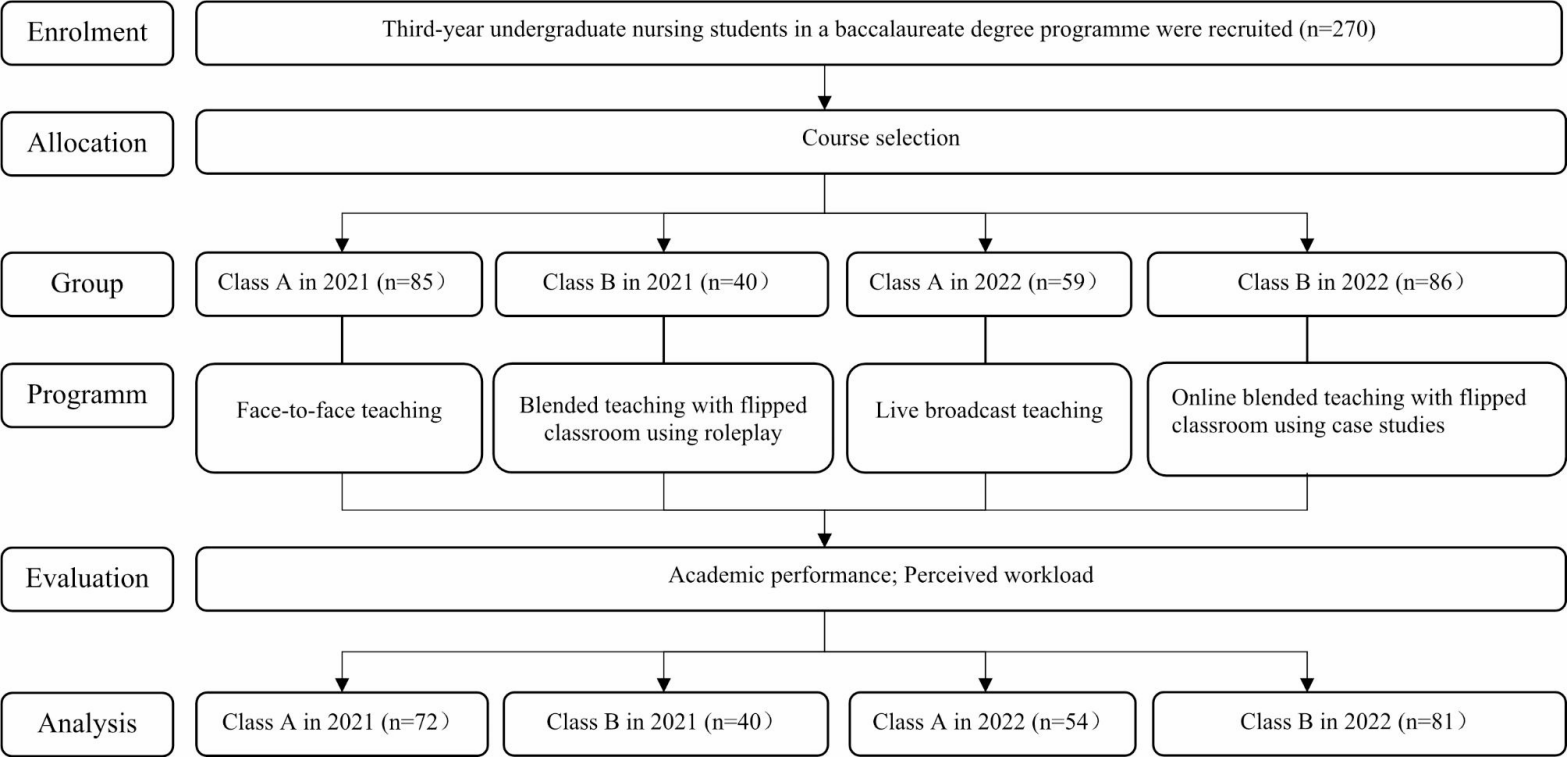
Flow diagram of the study (based on the CONSORT statement)

In total, 125 nursing students were enrolled in psychiatric nursing courses in 2021, and 145 nursing students were enrolled in 2022. The numbers of valid data points following the course exams and workload evaluations were 72 (84.71%) for Class A in 2021, 40 (100%) for Class B in 2021, 54 (91.53%) for Class A in 2022, and 81 (94.19%) for Class B in 2022. The flow diagram of the study is shown in Fig. 1. The average mean scores and SDs of participants’ GPAs before psychiatric nursing courses in the four teaching groups were 2.82 (SD 0.27), 2.85 (SD 0.26), 2.85 (SD 0.31), and 2.81 (SD 0.30), respectively. The total mean scores and SDs of participants’ academic performance in the four teaching groups were 25.28 (SD 4.34), 27.61 (SD 4.54), 28.59 (SD 3.10) and 29.85 (SD 3.95), respectively, and the total mean scores and SDs of perceived workload were 58.83 (SD 11.10), 47.51 (SD 13.55), 53.80 (SD 16.40) and 54.45 (SD 13.92), respectively. The sociodemographic characteristics of the participants are shown in Table 2.

**Table 2:** Descriptive statistics of the variables of the four teaching approach groups

Characteristics	Categories	Class A in 2021 (n = 72)	Class B in 2021 (n = 40)	Class A in 2022 (n = 54)	Class B in 2022 (n = 81)	*F/χ* ^*2*^	*P*
Age in years (Mean SD)		20.07 ± 0.83	20.04 ± 0.39	20.04 ± 0.78	20.14 ± 0.91	0.321	0.810
Gender (%)	Male	15(20.8)	0(0.00)	6(11.11)	16(19.75)	8.292	0.040^*^
	Female	57(79.17)	40(100.00)	48(88.89)	65(70.25)		
Birthplace (%)	Town	41(56.94)	36(90.00)	41(75.93)	41(50.62)	23.012	< 0.001^***^
	Rural area	31(43.56)	4(10.00)	13(24.07)	40(49.38)		
Ethnicity	Han	64	35	48	69	0.616	0.893
	Minority	8	5	6	12		
Experience interacting with persons with mental disorders, yes (%)	Yes	23(31.94)	6(15.00)	21(38.89)	34(41.98)	9.440	0.024^*^
	No	49(68.06)	34(85.00)	33(61.11)	47(58.01)		
History of personal mental health problems, yes (%)	Yes	17(23.61)	7(17.50)	9(16.67)	29(35.80)	8.292	0.040^*^
	No	55(76.39)	33(82.50)	45(83.33)	52(64.20)		
Grade point average		2.82 ± 0.27	2.85 ± 0.26	2.85 ± 0.31	2.81 ± 0.30	0.385	0.764
Academic performance		25.28 ± 4.34	27.61 ± 4.54	28.59 ± 3.10	29.85 ± 3.95	17.655	< 0.001^***^
Perceived workload		58.83 ± 11.10	47.51 ± 13.55	53.80 ± 16.40	54.45 ± 13.92	5.907	< 0.001^***^
	Mental demands	66.85 ± 18.70	57.53 ± 21.49	60.09 ± 20.01	64.07 ± 18.94	2.457	0.064
	Physical demands	44.94 ± 19.76	37.63 ± 22.21	41.76 ± 24.77	39.88 ± 26.41	1.000	0.394
	Temporal demands	60.61 ± 16.36	48.38 ± 23.43	50.00 ± 26.37	53.83 ± 20.98	3.801	0.011^*^
	Performance	65.43 ± 19.17	38.93 ± 25.19	58.80 ± 29.27	57.28 ± 25.41	10.060	< 0.001^***^
	Effort	69.75 ± 15.96	67.05 ± 20.09	74.35 ± 21.59	71.67 ± 17.82	1.331	0.265
	Frustration	45.42 ± 24.60	35.58 ± 26.95	37.78 ± 27.23	40.00 ± 22.83	1.664	0.175

Note: ^*^P < 0.05, ^***^P < 0.001

### Comparisons of the four teaching approaches

#### Comparisons of the impacts of the four teaching approaches on academic performance

The enter linear regression method was used to identify the variables influencing academic performance (adjusted *R*^*2*^ = 0.264, *F* = 13.601, *P* < 0.001). The results indicated that compared with face-to-face teaching (Class A in 2021), live broadcast teaching (Class A in 2022) (regression coefficient *B* = 2.698, *P* < 0.001) and online blended teaching with flipped classroom using case studies (Class B in 2022) (*B* = 4.422, *P* < 0.001) were associated with significantly higher academic performance scores.

**Fig. 2:**
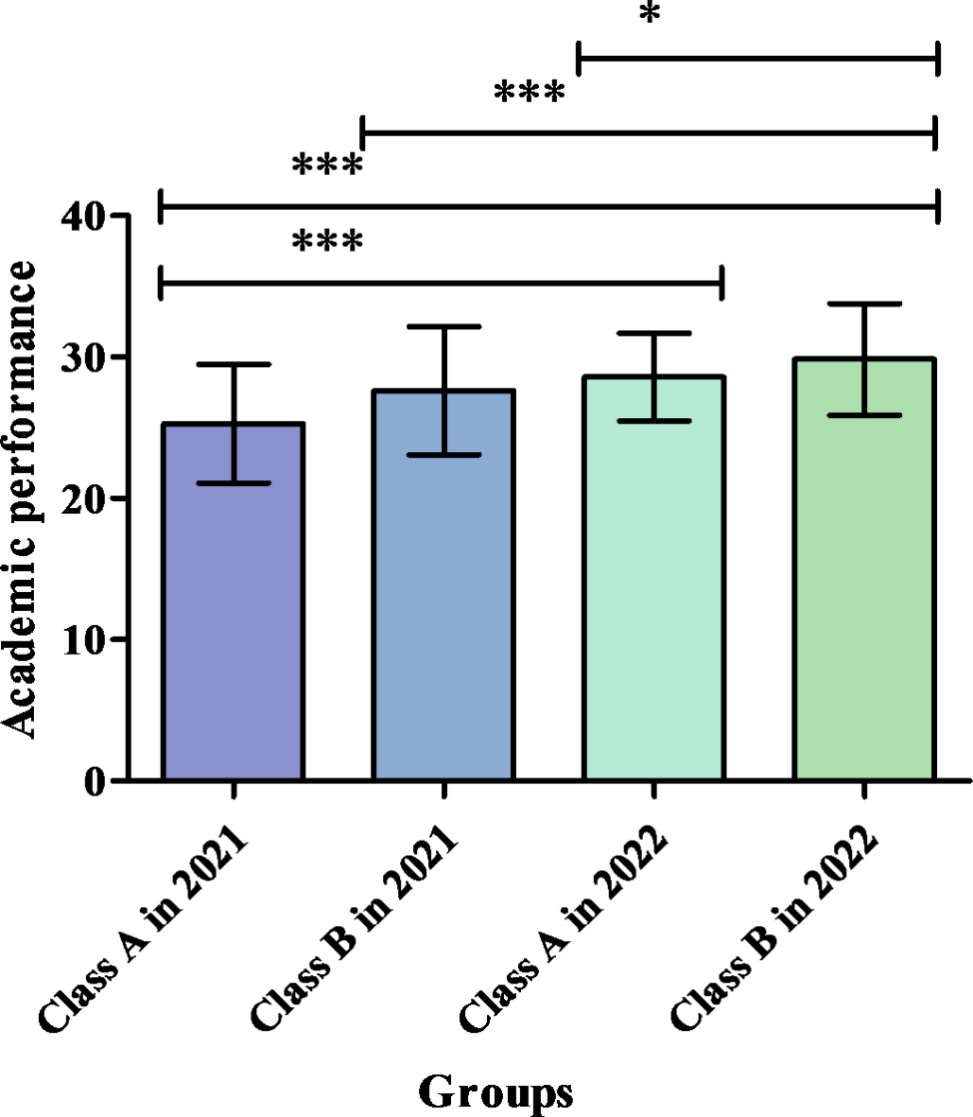
Comparisons of the impacts of the four teaching approaches on academic performance (Note: *P < 0.05, ***P < 0.001)

Furthermore, an analysis of the difference between the innovative groups differentiated by online synchronous and blended teaching revealed that compared with live broadcast teaching (Class A in 2022), online blended teaching with flipped classroom using case studies (Class B in 2022) (*B* = 2.539, *P* = 0.012) was associated with significantly higher academic performance scores, whereas blended teaching with flipped classroom using roleplay (Class B in 2021) (*B*=-1.734, *P* < 0.083) was not associated with any significant differences. The characteristics of academic performance and the comparisons among the four teaching approaches are presented in Fig. 2 and in Supplementary Tables 1 and 2.

#### Comparisons of the impacts of the four teaching approaches on perceived course workload

The enter linear regression method was used to identify the variables influencing perceived workload (adjusted *R*^*2*^ = 0.055, *F* = 3.047, *P* = 0.004). Linear regression analysis revealed that compared with face-to-face teaching (Class A in 2021), blended teaching with flipped classroom using roleplay (Class B in 2021) (regression coefficient *B*=-10.698, *P* < 0.001) was associated with a significantly lower level of perceived workload, and online blended teaching with flipped classroom using case studies (Class B in 2022) (*B*=-1.930, *P* = 0.055) was associated with a critical statistical difference in this context.

**Fig. 3:**
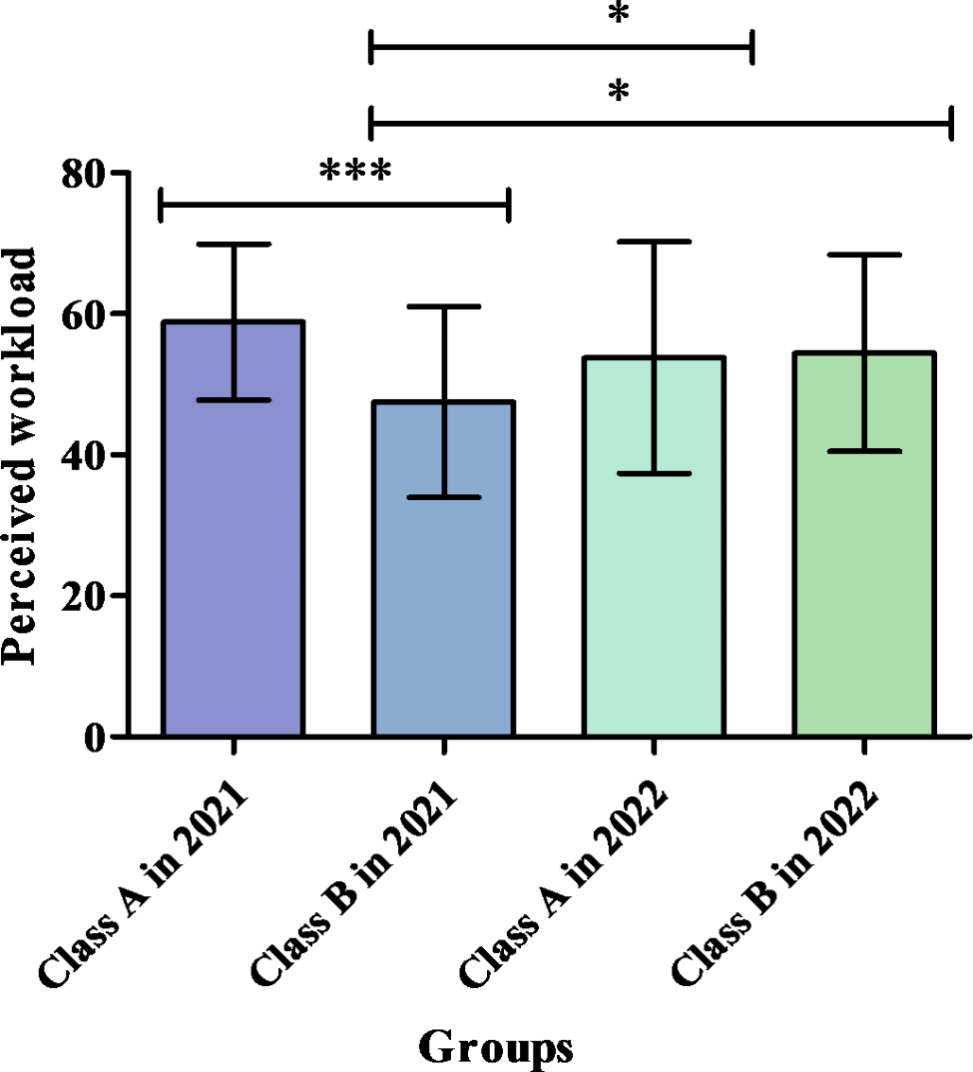
Comparisons of the impacts of the four teaching approaches on perceived course workload (Note: *P < 0.05, ***P < 0.001)

Furthermore, an analysis of the difference between the innovative groups revealed that compared with live broadcast teaching (Class A in 2022), blended teaching with flipped classroom using roleplay (Class B in 2021) was associated with the lowest level of perceived workload (*B*=-2.126, *P* = 0.034), whereas online blended teaching with flipped classroom using case studies (Class B in 2022) (*B* = 0.053, *P* = 0.958) was not associated with any significant differences. The characteristics of perceived workload and the comparisons among the four teaching approaches are presented in Fig. 3 and in Supplementary Tables 3 and 4.

## Discussion

### Interpreting the findings

Nursing education has recently undergone a shift in terms of teaching philosophies from lecture-based teaching towards student-centred learning with the goals of encouraging constructive learning and promoting learning engagement. Furthermore, due to the rapid development of digital technology, online education provides a pathway for distance learning. A single teaching method, such as problem-based learning, has been proven to be effective in facilitating students’ performance [24]. The impact of innovative teaching designs for synchronous, asynchronous and blended teaching warrants further investigation because during the COVID-19 pandemic, it became necessary for nursing curricula to adapt constantly to changes in the pandemic conditions by adopting new and hybrid education approaches without a foundation of targeted evidence [25]. To the best of the researchers’ knowledge, this study is the first to explore the major teaching approaches used in nursing education during the COVID-19 pandemic. These approaches were differentiated from each other based on whether they employed the principle of student-centred learning and the method of distance teaching. This study showed that the integration of online teaching with live broadcasts and case studies was the most effective method for improving students’ performance scores and that blended teaching with flipped classroom using roleplay was perceived as being associated with the lowest workload and thus encouraging students’ engagement.

According to the conventional view, the information transmission pathway is the only difference between traditional face-to-face pedagogy and live broadcast teaching. However, the comparison of the different approaches in terms of their influence on academic performance revealed that live broadcast teaching offered a significant advantage with regard to improving students’ academic scores, which is in line with the findings reported by Fang and colleagues [26]. As investigation of undergraduate nursing students has already shown that students’ perceptions of digital technology are positive, and so distance learning may offer further advantages, such as overcoming the “time and space limit” and thereby reducing task load in course learning [27]. This result shows that although face-to-face pedagogy and live broadcast teaching are synchronous teaching models, students in the digital age embrace and benefit from a virtual learning environment. However, live broadcast teaching is generally utilized as a temporary solution to meet the urgent need for distance learning or is applied within a laboratory setting [9] or clinical setting [27] to offer a clear angle of clinical observation, avoid interfering with clinicians, and reduce infection rates during surgical operations. Therefore, live broadcast teaching can usually be optimized by employing advanced designs such as blended teaching.

Consequently, as students were separated during some of the learning process due to social distancing, the teaching of the psychiatric nursing course was designed to feature a combination of face-to-face teaching, technology-mediated instruction and flipped classroom using roleplay (Class B in 2021); this approach was in line with the definition of blended learning [28]. The statistical analyses conducted in this study indicated a significant decrease in the perceived course workload without compromising the academic performance of students in this blended learning group. An explanation for this result can be found in the literature, which has claimed that flipped instruction emphasizes student-centred learning instead of one-way instructional learning because the former involves online self-directed learning and classroom activities, which increase the opportunities for collaborative learning and competency-based education practices available to students [11]. Furthermore, through roleplay exercises and/or team-based case studies, students construct and reconstruct knowledge through active participation and critical thinking, which promotes their learning interest and engagement and thus reduces their perceived workload [5]. Previous studies have highlighted the positive impact of blended learning on students’ academic success [29], especially the fact that the incorporation of self-directed learning into blended teaching design could improve vital competencies in the contemporary medical profession, such as lifelong learning skills and autonomous and exploratory characteristics [24, 30]. However, this study did not indicate significant academic improvement in this context. Possible explanations of this result are that roleplay exercises require excessive amounts of learning time and that the focus of the course may shift away from the learning objectives without proper guidance, as the learning content pertaining to psychiatric nursing is abstract and complex [5].

However, another approach to online blended learning used in this study offered a significant advantage in terms of students’ academic performance; this approach included live broadcasting pertaining to difficult content, self-directed learning and a problem-based case study. Compared with the academic performance improvement associated with the blended learning of Class B in 2021, of the improvement associated with the approach used for Class B in 2022 might have been due to the flexible online learning format and the fact that fewer roleplay exercises were included. However, Class B in 2022 exhibited an increased level of perceived workload, once again highlighting the importance of class immersion [5]. Overall, Class B in 2022 attained the highest level of performance with a moderate level of perceived workload, thus highlighting the feasibility, suitability and effectiveness of this approach during the pandemic. Accordingly, the approach used for Class B in 2022 was the optimal teaching approach in the context of distance learning. This finding has also been reported by relevant studies, showing that problem-based learning or team-based learning approaches can facilitate performance by increasing study time and learning engagement [31]. As the concepts of psychiatric nursing are abstract and complex, self-directed learning and the flipped classroom approach using problem-based learning provide an opportunity for students to explore concepts prior to and during classroom learning [32].

In this study, the between-group difference in academic performance and perceived workload might have been due to the inclusion of different flipped learning methods in the two blended learning designs. During the period of isolation due to the epidemic, the classroom activities involved in the traditional flipped learning method (Class B in 2021) in this study were replaced by the “untact” flipped learning model using a problem-based webinar case study (Class B in 2022), leading to further improvements in academic performance scores. However, the results of this study are inconsistent with those of a study that aimed to evaluate the effects of the contact and “untact” designs for flipped learning in a clinical internship setting [33]. This inconsistency might indicate that roleplay exercises may not be successful in improving academic performance in the context of psychiatric nursing education. This result is consistent with the conclusion of a recent review claiming that clinical roleplaying and reflection can enhance student involvement and self-efficacy in mental health practice without significantly improving academic scores [34]. As noted in the literature, other types of nursing simulation, such as standardized patients and high-fidelity simulation, appear to be effective with regard to enhancing psychiatric nursing skills and decision making [4], thus highlighting their possible applicability in the flipped classroom [35]. However, simulation teaching faces several challenges in a constantly changing teaching context, including the cost and time required to develop simulation technology and equipment, the lack of resources for large-scale teaching, and the inapplicability of this approach to distance teaching or the need for additional permissions when accessing online resources; these challenges may hinder the implementation of such simulation teaching [4, 36]. Therefore, the teaching design used for Class B in 2022, which was based on the flipped learning method via an online medium, is recommended by this study.

### Implications

According to the results of this study, several implications for psychiatric nursing education are proposed to provide further guidance for both research and practice.

#### Adopting student-centred constructive learning is the core principle of teaching design

In this study, the two blended learning approaches were superior to lecture-based didactics in terms of their impacts on both academic performance and perceived workload, thus highlighting the importance of student-centred constructive learning in teaching design. Moreover, balancing time commitments, reducing restrictions on the physical learning environment and optimizing teaching activities to promote students’ interest and involvement in course learning are essential to the success of teaching.

#### Appropriate and feasible education in psychiatric nursing is essential in terms of teaching contexts

In this study, four teaching approaches were used during the COVID-19 pandemic, and several elements were considered to ensure the appropriateness and feasibility of the courses, including the characteristics of psychiatric nursing, students’ preferences and local policies concerning pandemic control. In terms of the characteristics of psychiatric nursing, teaching abstract concepts to students is challenging; thus, didactic teaching in the form of lectures that are either held face-to-face, live-streamed or recorded should be supplemented with multimedia technology. Therefore, a SPOC focused on psychiatric nursing was presented on a digital platform and made accessible to all the students who participated in this study. In addition, as some of the major teaching objectives of psychiatric nursing are to facilitate students’ development of therapeutic communication skills, foster empathy, and diminish prejudice towards persons with mental disorders [37], practical education conducted using methods such as clinical practice, simulation training, and/or problem-based case studies could be an effective component of teaching success. Regarding teaching resources, including time, cost, the physical environment, equipment and software, roleplay exercises and problem-based case studies were used as the major methods of teaching in the flipped classroom during the pandemic; online broadcasts for clinical practice were also used in this study. However, the results might indicate that such roleplay exercises may not be more effective in the context of flipped teaching than problem-based case studies but that roleplay could nonetheless be an effective way of optimizing students’ perceived workload. Therefore, innovative ways to deliver flipped teaching by employing other simulation techniques, such as the development of high-fidelity patient simulation scenarios, might be needed in this context [35].

### Ethical considerations pertaining to online practical teaching in psychiatric nursing should be considered

It is well known that during the COVID-19 pandemic, clinical practice could be difficult and stressful for students and instructors, as it might increase the risk of infection faced by both students and patients. Therefore, online practical teaching has become an essential component of clinical education in cases in which distance learning is necessary. However, due to the wide connectivity facilitated by the internet, the rapid speed of information transfer may increase the risk of legal breaches, such as violations of patient privacy [11]. Therefore, online classroom discipline and network supervision are necessary when conducting online psychiatric nursing practice.

### Limitations

The authors acknowledge several limitations of this study. (1) A convenience sampling approach without a randomized controlled trial design in a single university was used, which might have limited the generalizability of the conclusions. However, the naturalistic educational setting of the study might have improved the generalizability of the data to other nursing educational settings to some degree. (2) Regarding the comparative indicators, the measurement of academic performance did not include the component of practical skills because policies pertaining to isolation in response to COVID-19 hindered clinical practice and skill evaluations during the time the study was conducted. In addition, the use of self-reported measures for perceived workload might have resulted in common method variance and social desirability bias.

## Conclusions

In this study on the effectiveness of four teaching approaches in the context of a psychiatric nursing course, the two blended learning approaches were found to be superior to lecture-based didactics. A blended learning teaching design that employs the flipped learning method using case studies conducted via an online medium is recommended during the pandemic due to its accessibility, feasibility, acceptability and effectiveness. Psychiatric nursing educators should be innovative, able to inspire and motivate students’ learning engagement and capable of balancing the learning workload, which could enhance students’ academic performance.

### Electronic supplementary material

Below is the link to the electronic supplementary material.

**Supplementary Table 1**. Analysis of the effects of teaching approaches on academic performance compared with Class A in 2021 using multivariable linear regression. **Supplementary Table 2**. Analysis of the effects of teaching approaches on academic performance compared with Class A in 2022 using multivariable linear regression. **Supplementary Table 3**. Analysis of the effects of teaching approaches on perceived workload compared with Class A in 2021 using multivariable linear regression. **Supplementary Table 4**. Analysis of the effects of teaching approaches on perceived workload compared with Class A in 2022 using multivariable linear regression.

## Data Availability

The datasets used and/or analysed during the current study are available from the corresponding author upon reasonable request.

## List of abbreviations

MOOC: Massive open online course
SPOCs: Small private online courses
GPA: Grade point average
NASA-TLX: National Aeronautics and Space Administration Task Load Index

## Declarations

### Guidelines statement

All methods were implemented in accordance with the relevant guidelines and regulations.
