# Supplementary material for: Innovations in teaching during the COVID-19 pandemic: comparisons of the impacts of different teaching approaches in psychiatric nursing on undergraduate nursing students
Source: BMC Med Educ. 2023 Nov 3;23:827. doi: 10.1186/s12909-023-04819-8 (PMC10623829; doi:10.1186/s12909-023-04819-8)
Supplement: Supplementary file 1 — Supplementary Table 1. Analysis of the effects of teaching approaches on academic performance compared with Class A in 2021 using multivariable linear regression. Supplementary Table 2. Analysis of the effects of teaching approaches on academic performance compared with Class A in 2022 using multivariable linear regression. Supplementary Table 3. Analysis of the effects of teaching approaches on perceived workload compared with Class A in 2021 using multivariable linear regression. Supplementary Table 4. Analysis of the effects of teaching approaches on perceived workload compared with Class A in 2022 using multivariable linear regression. [file 12909_2023_4819_MOESM1_ESM.docx]

| Supplementary Table 1. Analysis of the effects of teaching approaches on academic performance compared with Class A in 2021 using multivariable linear regression | | | | | | |
| --- | --- | --- | --- | --- | --- | --- |
| Variables | Unstandardized coefficients B | Standardized coefficients β | *t* | *P* | 95% CI for B | |
|  |  |  |  |  | Lower Bound | Upper bound |
| Constant | 26.282 |  | 45.026 | 0.000 | 25.132 | 27.432 |
| Class A in 2022 | 2.698 | 0.257 | 3.868 | 0.000 | 1.324 | 4.072 |
| Class B in 2021 | 1.309 | 0.111 | 1.650 | 0.100 | -0.254 | 2.872 |
| Class B in 2022 | 4.422 | 0.479 | 7.260 | 0.000 | 3.222 | 5.622 |
| Gender (Male) | -3.888 | -0.320 | -5.713 | 0.000 | -5.228 | -2.547 |
| Birthplace (Rural area) | -0.633 | -0.071 | -1.211 | 0.227 | -1.664 | 0.397 |
| Experience interacting with persons with mental disorders (Yes) | 0.444 | 0.049 | 0.806 | 0.421 | -0.641 | 1.530 |
| History of personal mental health problems (Yes) | 0.105 | 0.010 | 0.175 | 0.861 | -1.073 | 1.282 |
| Note. Dependent Variable: academic performance; Model Summary: adjusted *R^2^* = 0.264, *F*= 13.601, *P*<0.001 | | | | | | |

| Supplementary Table 2. Analysis of the effects of teaching approaches on academic performance compared with Class A in 2022 using multivariable linear regression. | | | | | | |
| --- | --- | --- | --- | --- | --- | --- |
| Variables | Unstandardized coefficients B | Standardized coefficients β | *t* | *P* | 95% CI for B | |
|  |  |  |  |  | Lower Bound | Upper bound |
| Constant | 28.979 |  | 50.998 | 0.000 | 27.860 | 30.099 |
| Class B in 2021 | -1.389 | -0.118 | -1.743 | 0.083 | -2.958 | 0.181 |
| Class B in 2022 | 1.724 | 0.187 | 2.539 | 0.012 | 0.386 | 3.062 |
| Class A in 2021 | -2.698 | -0.283 | -3.868 | 0.000 | -4.072 | -1.324 |
| Gender (Male) | -3.888 | -0.320 | -5.713 | 0.000 | -5.228 | -2.547 |
| Birthplace (Rural area) | -0.633 | -0.071 | -1.211 | 0.227 | -1.664 | 0.397 |
| Experience interacting with persons with mental disorders (Yes) | 0.444 | 0.049 | 0.806 | 0.421 | -0.641 | 1.530 |
| History of personal mental health problems (Yes) | 0.105 | 0.010 | 0.175 | 0.861 | -1.073 | 1.282 |
| Note. Dependent Variable: academic performance; Model Summary: adjusted *R^2^* = 0.264, *F*= 13.601, *P*<0.001 | | | | | | |

| Supplementary Table 3. Analysis of the effects of teaching approaches on perceived workload compared with Class A in 2021 using multivariable linear regression | | | | | | |
| --- | --- | --- | --- | --- | --- | --- |
| Variables | Unstandardized coefficients B | Standardized coefficients β | *t* | *P* | 95% CI for B | |
|  |  |  |  |  | Lower Bound | Upper bound |
| Constant | 58.254 |  | 27.106 | 0.000 | 54.020 | 62.488 |
| Class A in 2022 | -4.462 | -0.131 | -1.738 | 0.084 | -9.521 | 0.597 |
| Class B in 2021 | -10.698 | -0.280 | -3.662 | 0.000 | -16.453 | -4.943 |
| Class B in 2022 | -4.329 | -0.144 | -1.930 | 0.055 | -8.746 | 0.089 |
| Gender (Male) | 4.280 | 0.108 | 1.708 | 0.089 | -0.656 | 9.216 |
| Birthplace (Rural area) | -0.135 | -0.005 | -0.070 | 0.944 | -3.929 | 3.660 |
| Experience interacting with persons with mental disorders (Yes) | -1.668 | -0.056 | -0.822 | 0.412 | -5.664 | 2.329 |
| History of personal mental health problems (Yes) | 1.258 | 0.039 | 0.572 | 0.568 | -3.076 | 5.592 |
| Note. Dependent Variable: perceived workload; Model Summary: adjusted *R^2^* = 0.055, *F*= 3.047, *P*=0.004 | | | | | | |

| Supplementary Table 4. Analysis of the effects of teaching approaches on perceived workload compared with Class A in 2022 using multivariable linear regression | | | | | | |
| --- | --- | --- | --- | --- | --- | --- |
| Variables | Unstandardized coefficients B | Standardized coefficients β | *t* | *P* | 95% CI for B | |
|  |  |  |  |  | Lower Bound | Upper bound |
| Constant | 53.792 |  | 25.711 | 0.000 | 49.671 | 57.914 |
| Class B in 2021 | -6.236 | -0.163 | -2.126 | 0.034 | -12.013 | -0.459 |
| Class B in 2022 | 0.133 | 0.004 | 0.053 | 0.958 | -4.793 | 5.060 |
| Class A in 2021 | 4.462 | 0.144 | 1.738 | 0.084 | -0.597 | 9.521 |
| Gender (Male) | 4.280 | 0.108 | 1.708 | 0.089 | -0.656 | 9.216 |
| Birthplace (Rural area) | -0.135 | -0.005 | -0.070 | 0.944 | -3.929 | 3.660 |
| Experience interacting with persons with mental disorders (Yes) | -1.668 | -0.056 | -0.822 | 0.412 | -5.664 | 2.329 |
| History of personal mental health problems (Yes) | 1.258 | 0.039 | 0.572 | 0.568 | -3.076 | 5.592 |
| Note. Dependent Variable: perceived workload; Model Summary: adjusted *R^2^* = 0.055, *F*= 3.047, *P*=0.004 | | | | | | |
